# The first case of human infection with H5N1 avian Influenza A virus in Chile

**DOI:** 10.1093/jtm/taad083

**Published:** 2023-06-13

**Authors:** Andrés Castillo, Rodrigo Fasce, Barbara Parra, Winston Andrade, Paulo Covarrubias, Andrea Hueche, Constanza Campano, Carolina Tambley, Marcelo Rojas, Maykol Araya, Felipe Hernández, Patricia Bustos, Jorge Fernández

**Affiliations:** Molecular Genetics Subdepartment, Public Health Institute of Chile, 1000 Ñuñoa, Chile; Section of Respiratory and Exanthematic Viruses, Institute of Public Health of Chile, 1000 Ñuñoa, Chile; Molecular Genetics Subdepartment, Public Health Institute of Chile, 1000 Ñuñoa, Chile; Section of Respiratory and Exanthematic Viruses, Institute of Public Health of Chile, 1000 Ñuñoa, Chile; Molecular Genetics Subdepartment, Public Health Institute of Chile, 1000 Ñuñoa, Chile; Section of Respiratory and Exanthematic Viruses, Institute of Public Health of Chile, 1000 Ñuñoa, Chile; Molecular Genetics Subdepartment, Public Health Institute of Chile, 1000 Ñuñoa, Chile; Section of Respiratory and Exanthematic Viruses, Institute of Public Health of Chile, 1000 Ñuñoa, Chile; Molecular Genetics Subdepartment, Public Health Institute of Chile, 1000 Ñuñoa, Chile; Clinical Laboratory, Regional Hospital of Antofagasta, 10255 Antofagasta, Chile; Section of Respiratory and Exanthematic Viruses, Institute of Public Health of Chile, 1000 Ñuñoa, Chile; Section of Respiratory and Exanthematic Viruses, Institute of Public Health of Chile, 1000 Ñuñoa, Chile; Molecular Genetics Subdepartment, Public Health Institute of Chile, 1000 Ñuñoa, Chile

**Keywords:** Avian influenza, H5N1, Chile, human case, clade 2.3.4.4b, Influenza A

## Abstract

Highlights

- Here we present the first human case of Influenza A H5N1 infection in Chile, and the fifth worldwide in 2023.
- The patient is a 53-year-old man who lives in the north region of Chile, near the seashore.
- The Chilean sample was subtyped in the clade 2.3.4.4b.

Avian influenza (AI), commonly named bird flu, is a type of Influenza virus that affects wild birds and poultry. The AI viruses can be present in a combination of 16 hemagglutinin (HA) and 9 neuraminidase (NA) subtypes. AI H5N1 cases were first described in Hong Kong in 1997 when birds died from an outbreak of the disease.^1^ Later that year, a zoonotic transmission of H5N1 was reported in a 3-year-old child in Hong Kong, the first case of H5N1 disease in humans. At that time, the outbreak affected 18 patients with a case fatality rate of 33% for this virus subtype.^2^^,^^3^

As of 5 March 2023, 873 cases of human infection with AI H5N1 have been reported globally, including 458 deaths.^4^ In 2023, this included four cases of zoonotic infections: one in Ecuador (2.3.4.4b clade), one in China (2.3.4.4b clade) and two in Cambodia (2.3.2.1c clade).^5^

The patient from Ecuador was described as a 3-year-old girl in contact with backyard poultry who died without an apparent cause, coinciding with reports of sudden deaths of backyard poultry in that province. This case was reported as the first case in the South American region.^6^

On March 29, 2023, Chilean authorities confirmed the first national case of human infection with AI H5N1. An epidemiological investigation being conducted by the Ministry of Health ascertained that the patient’s residence is located one block from the seashore where seabirds infected with H5N1 viruses had previously been detected. For that reason, an infection of environmental origin is suspected. The investigation is not yet complete as the patient remains hospitalised, but no new human cases of AI H5N1 have been reported in the country.

The confirmed case is a 53-year-old man who lives in the region of Antofagasta, in the north of Chile, with no comorbidities. When his initially mild symptoms of a cough and sore throat worsened, he was admitted on 22 March to the Regional Hospital of Antofagasta, which is one of the severe acute respiratory infections (SARI) surveillance sentinel centers in Chile.

An initial nasopharyngeal swab sample was negative for SARS-CoV-2 by RT-PCR. On 23 March, he was admitted to the intensive care unit. The next day the patient started treatment with oseltamivir and antibiotics. He remains in respiratory isolation under multidisciplinary management, with mechanical ventilation due to pneumonia. In the ICU, a bronchoalveolar sample was collected and tested in the hospital and a unsubtypeable Influenza A case was detected. As part of the surveillance protocols, the sample was referred for confirmation, viral subtyping analysis and NGS to the national influenza center, at the Public Health Institute (ISP) of Chile. At ISP, the sample was tested with both the CDC Influenza Virus Real-Time RT-PCR Influenza A (H3/H1pdm09) Subtyping kit (negative result for both subtypes and positive for Influenza A with Ct = 23) and with the CDC Influenza A/H5 Subtyping kit (Ct = 25).

The 8 fragments of the AI H5N1 genome were amplified by RT-PCR One-step. The sequencing was performed using 100 ng of the DNA using Nextera DNA Flex Library Prep Kit and paired-end sequencing (2 × 150 bp) an Illumina MiSeq, with about ~2MIL total reads. For Nanopore sequencing, 400-ng DNA was used for library preparation using Rapid Barcoding Kit and sequencing in MinION Mk1C with about ~ 100 k total reads. Reads were mapped to the reference Influenza A virus (A/Thailand/1(KAN-1)/2004(H5N1)) (GenBank accession:266827).

**Figure 1:**
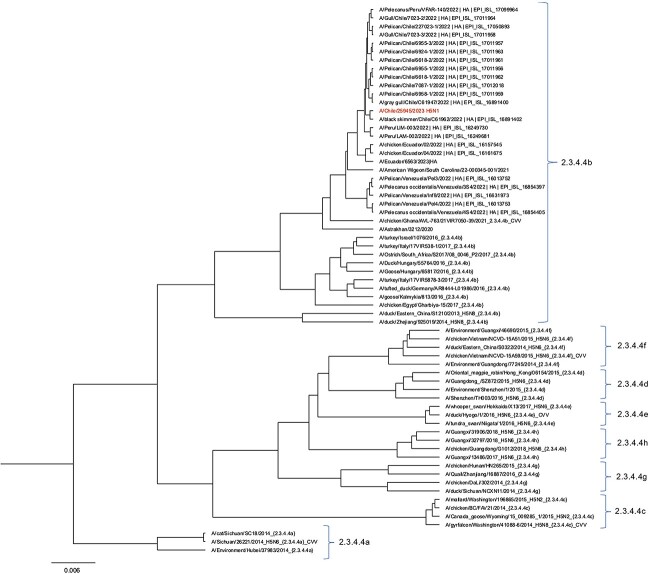
Maximum-Likelihood (GTR model) phylogenetic tree from HA fragment nucleotide sequence. Phylogenetic reconstruction was performed with 65 sequences selected from different origins, mainly Chile, South America and other regions of the world. The red highlighted sequence corresponds to the first Chilean human case HA sequence and shows the location within the 2.3.4.4b clade.

Haemagglutinin HA gene alignment of all selected sequences was performed using the MAFFT v7.407 multiple sequence alignment program. Through the IQ-TREE multicore v2.2.0, the best replacement model was selected, which was GTR + F + G4. The phylogenetic tree was built using the Beast V1.10.4 software, the Bayesian approximation using the Markov chain Monte Carlo (MCMC) method, and a non-parametric Bayesian Skyline (Piecewise-constant) coalescing model with a strict molecular clock method. A total of 20 million iterations were carried out for the alignment, which was subsampled every 1000 generations applying random selection as the initial tree. Along with this analysis, a Marginal Likelihood Estimation (MLE) using path sampling/stepping-stone sampling was performed and the maximum Clade Credibility tree was generated by discarding 20% of burnt-it. The resulting tree was visualised with Figtree v1.4.4.

The Chilean sample was subtyped in the clade 2.3.4.4b, like the other Latin American samples from Perú, Venezuela and Ecuador (Figure 1). The first Chilean case of human infection appears a few months after other AI H5N1 reports of infected Peruvian pelicans in Lluta river wetlands in December 2022, paired with infected sea lions and marine otters in the south of Peru (note: all of those viral isolates belong to the same clade 2.3.4.4b).^7^^,^^8^

Given that the reported cases of AI H5N1 in wild seabirds and sea mammals in the north of Chile has risen to 53 findings since March 1, it is vitally important to take educational measures, like community awareness and cautious behaviour before dead animals in the sea shore, also, expand the genomic surveillance to other marine species and poultry that could be vulnerable to zoonotic infections.^9^ Considering that the seasonal influenza season is approaching in the southern hemisphere, these types of outbreaks and zoonotic events put at risk and dangerously alert the possibility of latent recombination events between different types of influenza viruses in natural animal reservoirs and/or human populations.

## Data Availability

All the sequence data was uploaded to public databases.

## CRediT author statement

Andres E. Castillo (Conceptualization-Equal, Data curation-Equal, Formal analysis-Equal, Writing—original draft-Lead, Writing—review and editing-Lead), Rodrigo Fasce (Conceptualization-Equal, Data curation-Equal, Writing—original draft-Lead, Writing—review and editing-Lead), Barbara Parra (Conceptualization-Equal, Data curation-Equal), Winston Andrade (Conceptualization-Equal, Data curation-Equal), Paulo Covarrubias (Conceptualization-Equal, Data curation-Equal), Andrea Hueche (Conceptualization-Equal, Data curation-Equal), Constanza Campano (Conceptualization-Equal, Data curation-Equal, Formal analysis-Equal), Carolina Tambley (Conceptualization-Equal, Data curation-Equal), Marcelo Rojas (Conceptualization-Equal, Data curation-Equal, Formal analysis-Equal), Maykol Araya (Conceptualization-Equal, Data curation-Equal), Felipe Hernandez (Conceptualization-Equal, Data curation-Equal), Patricia Bustos (Conceptualization-Equal, Data curation-Equal), Jorge Fernandez (Conceptualization-Equal, Data curation-Equal, Writing—original draft-Lead, Writing—review and editing-Lead).
